# *AGO1* Homeostasis Involves Differential Production of 21-nt and 22-nt miR168 Species by *MIR168a* and *MIR168b*

**DOI:** 10.1371/journal.pone.0006442

**Published:** 2009-07-30

**Authors:** Hervé Vaucheret

**Affiliations:** Laboratoire de Biologie Cellulaire, Institut Jean-Pierre Bourgin, INRA, Versailles, France; University of Melbourne, Australia

## Abstract

**Background:**

AGO1 associates with microRNAs (miRNAs) and regulates mRNAs through cleavage and translational repression. AGO1 homeostasis entails DCL1-dependent production of miR168 from *MIR168a* and *MIR168b* transcripts, post-transcriptional stabilization of miR168 by AGO1, and AGO1-catalyzed miR168-guided cleavage of *AGO1* mRNA.

**Principal Findings:**

This study reveals that *MIR168a* is highly expressed and predominantly produces a 21-nt miR168 species. By contrast, *MIR168b* is expressed at low levels and produces an equal amount of 21- and 22-nt miR168 species. Only the 21-nt miR168 is preferentially stabilized by AGO1, and consequently, the accumulation of the 22-nt but not the 21-nt miR168 is reduced when DCL1 activity is impaired. *mir168a* mutants with strongly reduced levels of 21-nt miR168 are viable but exhibit developmental defects, particularly during environmentally challenging conditions.

**Conclusions/Significance:**

These results suggest that 22-nt miR168 ensures basal cleavage of *AGO1* mRNA whereas 21-nt miR168 permits an effective response to endogenous or environmental fluctuations owing to its preferential stabilization by AGO1.

## Introduction

20- to 30-nt long small RNAs regulate gene expression by guiding transcriptional silencing, mRNA cleavage and translation repression of homologous target genes [1], [2]. MicroRNAs (miRNAs) are produced by the precise excision of an approximately 21-nt miRNA/miRNA* duplex from the stem of a single-stranded, stem-loop RNA precursor [3]. Short interfering RNAs (siRNAs) derive from long perfect double-stranded RNAs (dsRNAs) that result from convergent transcription or transformation of single-stranded RNA into dsRNA by RNA-dependent RNA polymerases [4]. PIWI-associated RNAs (piRNAs) are produced by the shortening of a long single-stranded precursor RNA [5]. A common characteristic of all types of small RNAs is their association with proteins of the ARGONAUTE family, which is comprised of both AGO and PIWI members [6], [7]. The identity of the associated AGO protein determines the functional output of the associated small RNA (transcriptional silencing, mRNA cleavage or translation repression). Plants have miRNAs and siRNAs but no piRNAs. Consistently, plants encode AGO proteins but no PIWI proteins. The model plant species *Arabidopsis thaliana* potentially encodes 10 AGO proteins [7]. AGO1 catalyzes broad miRNA- and siRNA-guided mRNA cleavage and translation repression [8], [9], [10], [11]. AGO10 appears to be limited to miRNA- and siRNA-guided translation repression [9]. AGO7 specifically associates with miR390 and cleaves *TRANS-ACTING siRNA3* (*TAS3*) RNA to set the nucleotide frame for tasiRNA production, but the basis of this specific association is unknown [12]. AGO4 and AGO6 associate with endogenous siRNAs that are 24-nt long and guide transcriptional silencing [13], [14], [15], [16]. The functions of the five other Arabidopsis AGO proteins remain unknown.

AGO1-catalyzed miRNA-directed mRNA cleavage tolerates some mismatches, particularly near the miRNA 3′ end of the miRNA/mRNA duplex [17], [18]. Consequently, a given miRNA can target mRNAs that differ slightly in their targeted sequences [19]. However, these targets generally are members of a multigene family. Reciprocally, a given mRNA can be targeted by multiple miRNAs that differ only slightly in their sequences and, as such, derive from members of a *MIR* multigene family. Indeed, while most non-conserved miRNAs derive from a single locus, conserved miRNAs commonly are produced from multiple *MIR* loci [20], [21]. Therefore assigning the contribution of each *MIR* locus to the total miRNA pool and to target regulation often is difficult. So far, only a handful of phenotypic *mir* single mutants have been described, including *Petunia hybrida bl/mir169*, *Antirrhinum majus fis/mir169*, *Zea mays ts4/mir172e*, and *Arabidopsis thaliana mir164a, miR164b* and *mir164c* [22], [23], [24], [25], [26].

AGO1 is both an essential actor and a target of miRNA and siRNA pathways. Indeed, the level of *AGO1* mRNA is regulated by both the miRNA miR168 and by siRNAs generated from the *AGO1* mRNA after miR168-mediated cleavage [11], [27]. In addition, we previously reported that *AGO1* and *MIR168a* are transcriptionally co-regulated, which together with the coordinated action of miR168-guided *AGO1* mRNA cleavage and AGO1-mediated stabilization of miR168, keeps AGO1 levels in check [11], [28]. However, miR168 is produced from two loci, *MIR168a* and *MIR168b*, evoking questions about the respective contributions of these two loci. Here, we examine the roles of the *MIR168a* and *MIR168b* loci and show that they produce both 21-nt and 22-nt miR168 species. While both the 21-nt and 22-nt miR168 contribute to basal *AGO1* mRNA cleavage, the preferential stabilization of 21-nt miR168 by AGO1 protein likely provides a layer of adaptive response to endogenous or environmental fluctuations.

## Results

### Both *4m-MIR168a* and *4m-MIR168b* constructs rescue the *4m-AGO1* phenotype

**Figure 1:**
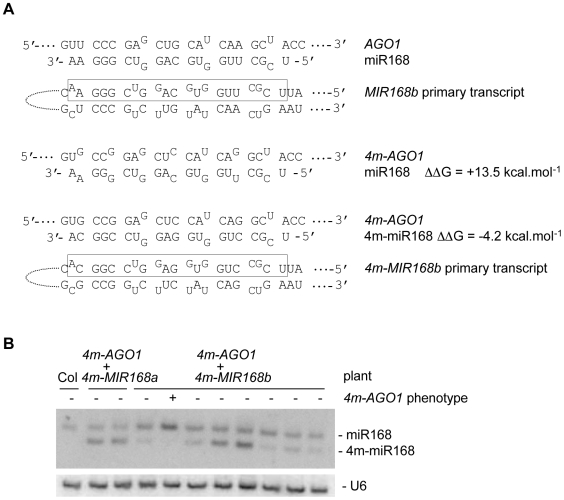
Compensatory mutations in the *MIR168b* gene rescue developmental defects induced by silent mutations in the miR168 complementary site of the *AGO1* mRNA nearly as efficiently as compensatory mutations in the *MIR168a* gene. (A) The *MIR168b* gene encodes a primary transcript that is partially paired (unpaired nucleotides are in superscript). The miRNA is boxed. Compensatory mutations in the *4m-MIR168b* transgene conserved the structure of the primary transcript and restored pairing with the *4m-AGO1* mRNA. Original mismatches were kept. ΔΔG was calculated using mfold. (B) Accumulation of the compensatory miRNA (4m-miR168) in plants transformed with *4m-AGO1* and either *4m-MIR168a* or *4m-MIR168b*. RNA gel blot analysis was performed using 20 µg of total RNA extracted from non-transformed plants (Col) and independent double transformants. The blot was first hybridized with a probe complementary to 4m-miR168, and re-hybridized with a probe complementary to miR168. U6 serves as a loading control.

We previously reported that introducing an *AGO1* mutant construct (*4m-AGO1*) carrying four silent mutations in the miR168 complementarity site in Arabidopsis results in a series of developmental defects that can be rescued by co-introducing a *MIR168a* mutant construct (*4m-MIR168a*) carrying compensatory mutations that produces a miRNA (4m-miR168) that can pair with the *4m-AGO1* mRNA [11]. These results indicate that the *MIR168a* gene is functional and sufficient for the proper regulation of its target gene *AGO1*. To determine whether the *MIR168b* gene, which also produces miR168, is functionally redundant with *MIR168a* or if *MIR168a* is the only active gene, the genomic region corresponding to the *MIR168b* gene, i.e. the segment of DNA comprised between the two adjacent genes, was mutagenized to introduce compensatory mutations (Figure 1A) and wildtype Arabidopsis plants were co-transformed with the *4m-AGO1* construct and either the *4m-MIR168a* or *4m-MIR168b* construct. Whereas 18 out of 27 *4m-AGO1* transformants displayed the *4m-AGO1* phenotype, only five out of 39 *4m-AGO1/4m-MIR168a* co-transformants and six out of 46 *4m-AGO1/4m-MIR168b* co-transformants displayed this phenotype. Double transformants that exhibited a wildtype phenotype accumulated detectable levels of 4m-miR168, which migrated faster than miR168 (Figure 1B). In contrast, 4m-miR168 was undetectable in double *4m-AGO1/4m-MIR168b* transformants that exhibited the *4m-AGO1*phenotype, and the endogenous miR168 level was increased in these plants (Figure 1B lane 5), consistent with previous observations in phenotypic *4m-AGO1* plants [28]. Since both *4m-MIR168b* and *4m-MIR168a* constructs are expressed under the control of their own regulatory elements, these results suggest that both the *MIR168a* and *MIR168b* genes are capable of regulating *AGO1* mRNA. Nevertheless, 4m-miR168 accumulation generally was lower than miR168 in *4m-AGO1/4m-MIR168b* plants (Figure 1B) whereas there accumulation was similar in *4m-AGO1/4m-MIR168a* plants [11]. This suggests that the *MIR168b* gene is expressed at a level lower than that of the *MIR168a* gene, consistent with miR168b* being less abundant than miR168a* in wildtype plants (Table 1 and [20]).

**Table 1:** Cloning frequency of miR168 species.

miRNA species	Locus origin	Sequence (5′to 3′)	Cloning frequency
22-nt miR168	MIR168a/b	TCGCTTGGTGCAGGTCGGGAAC	254
21-nt miR168	MIR168a/b	TCGCTTGGTGCAGGTCGGGAA	5039
22-nt miR168a*	MIR168a	TCCCGCCTTGCATCAACTGAAT	59
21-nt miR168a*	MIR168a	CCCGCCTTGCATCAACTGAAT	1912
22-nt miR168b*	MIR168b	TCCCGTCTTGTATCAACTGAAT	15
21-nt miR168b*	MIR168b	CCCGTCTTGTATCAACTGAAT	63

Cloning frequency indicates the number of times each small RNA sequence was cloned among a population of 887,000 reads [20].

### The *MIR168b* promoter has more limited expression than the *MIR168a* promoter

**Figure 2:**
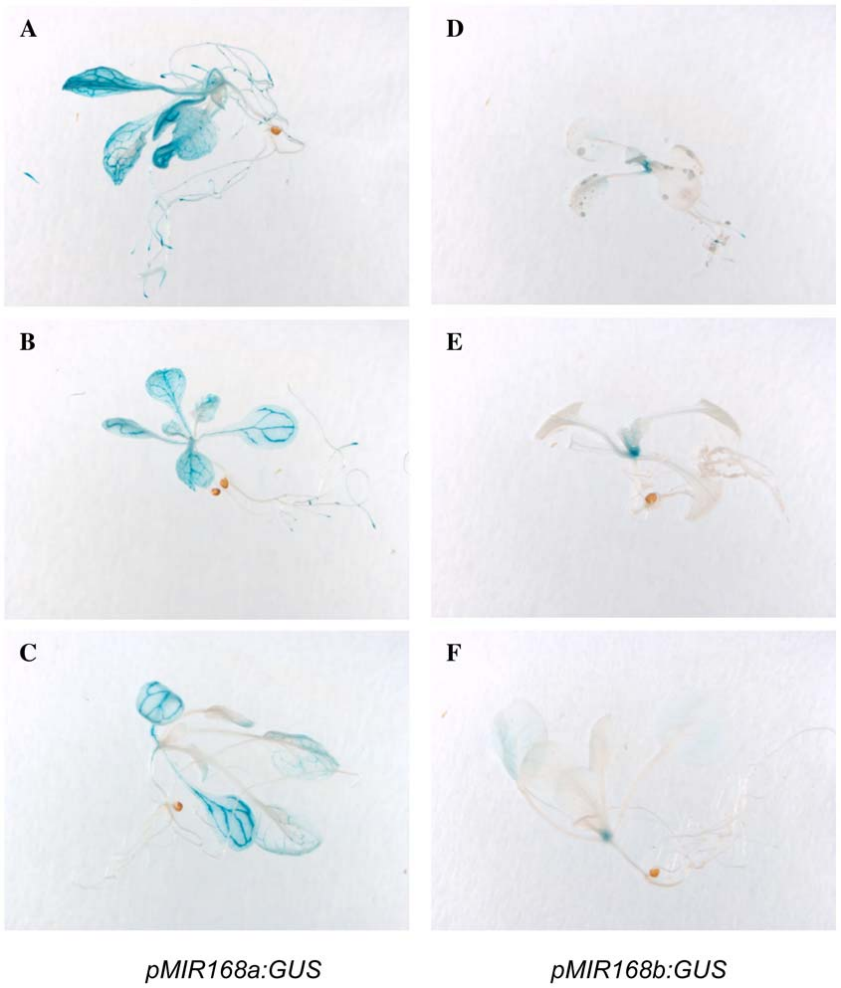
Expression from the *MIR168a* promoter is stronger and broader than that of the *MIR168b* promoter. The promoters of the *MIR168a* and *MIR168b* genes were fused upstream of the *uidA* reporter gene encoding GUS, and the resulting transgenes were introduced into wildtype Arabidopsis plants. Over-night X-gluc staining of three representative *pMIR168a∶GUS* (A–C) and *pMIR168b∶GUS* (D–F) transformants is shown (blue color indicates the presence of GUS).

To determine *MIR168a* and *MIR168b* promoter strengths and expression patterns, the 5′ sequences of the *MIR168a* and *MIR168b* genes, i.e. the genomic region comprised between the miR168 hairpins and the upstream adjacent genes, were fused upstream of the *uidA* reporter gene encoding GUS, and the resulting transgenes were introduced into wildtype Arabidopsis plants. The *MIR168a* promoter was subcloned as a 1339 bp fragment (position −1358 to −19 relative to the beginning of the hairpin), to remove an ATG located at position −15 that could compromise the use of the GUS reporter initiation codon and to keep the entire promoter region previously used to express the compensatory miRNA 4m-miR168 from the *4m-MIR168a* transgene [11], [28]. Similarly, the *MIR168b* promoter was cloned as a 479 bp fragment (position −516–37 relative to the beginning of the hairpin) to remove an ATG located at position −29 and to keep the promoter region that is sufficient to express the compensatory miRNA 4m-miR168 from the *4m-MIR168b* transgene (Figure 1A). Among 94 *pMIR168a∶GUS* transformants, 91 exhibited detectable GUS expression, ranging from faint to strong GUS staining as expected from T-DNA insertion positions affecting transgene expression. In contrast, only 24 out of 83 *pMIR168b∶GUS* transformants had detectable GUS expression, suggesting that the strength of the *pMIR168b* promoter is much lower than that of the *pMIR168a* promoter, consistent with the lower cloning frequency of miR168b* versus miR168a* (Table 1 and [20]). GUS expression was detected in shoot and root apical meristems of both *pMIR168a∶GUS* and *pMIR168b∶GUS* transformants whereas GUS expression in leaves was more restricted from *pMIR168b∶GUS* compared with *pMIR168a∶GUS* (Figure 2). It is unlikely that the lower and restricted expression of the *pMIR168b∶GUS* construct is due to a lack of regulatory elements of the *MIR168b* promoter because constructs carrying longer versions of the promoter, i.e. up to position −737 or −1520 relative to the beginning of the hairpin, exhibited similar patterns of GUS expression [29]. Together, these results indicate that expression from the *MIR168a* promoter is stronger and broader than that from the *MIR168b* promoter.

### *MIR168a* and *MIR168b* loci produce distinct amounts of 21- and 22-nt miRNA

**Figure 3:**
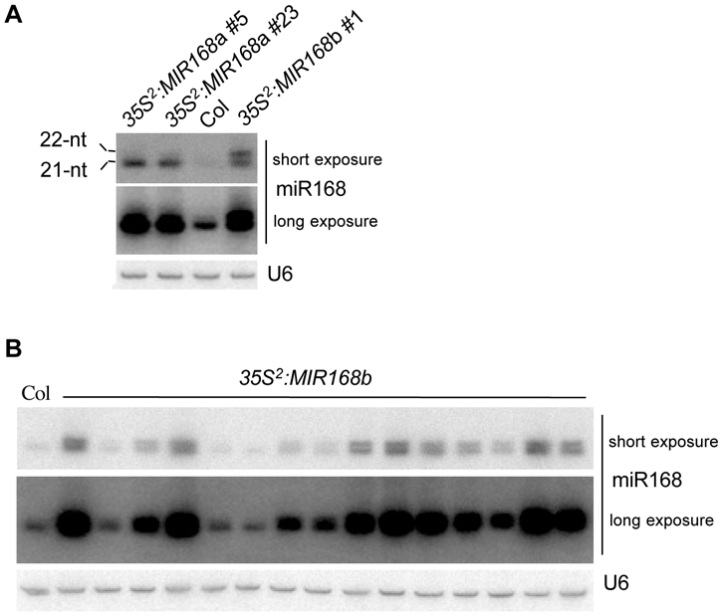
A 22-nt miR168 species accumulates in *35S^2^∶MIR168b* plants but not in *35S^2^∶MIR168a* plants. (A) miR168 accumulation in untransformed Col and individual *35S^2^∶MIR168a* and *35S^2^∶MIR168b* transformants generated using the binary vector pBin+. RNA gel blot analysis of 10 µg of RNA with a probe complementary to miR168. U6 serves as a loading control. (B) miR168 accumulation in untransformed Col and 15 individual *35S^2^∶MIR168b* transformants generated using the binary vector pCambia1200. RNA gel blot analysis of 10 µg of RNA hybridized with a probe complementary to miR168. U6 serves as a loading control.

To further examine the contributions of the *MIR168a* and *MIR168b* genes, the *MIR168a* and *MIR168b* stem-loops were expressed under the control of the 35S promoter in wildtype Arabidopsis plants. As expected from the T-DNA insertion position affecting transgene expression and as previously reported for the *35S^2^∶MIR168a* construct [28], *35S^2^∶MIR168b* transformants accumulated miR168 at varying levels, with those plants accumulating high levels of miR168 exhibiting large and serrated leaves and flowering late compared with wildtype plants (data not shown), indicating that both *35S^2^∶MIR168a* and *35S^2^∶MIR168b* constructs are functional. However, in contrast to *35S^2^∶MIR168a* transformants that over-accumulated a miR168 species that co-migrated with the endogenous 21-nt miR168 [28], *35S^2^∶MIR168b* transformants over-accumulated equal amounts of 21-nt and 22-nt miR168 species (Figure 3A and data not shown). To rule out a construct artifact, the *35S^2^∶MIR168b* construct was rebuilt into a different binary vector and introduced into wildtype Arabidopsis. Analysis of a series of 15 transformants confirmed in plants accumulating miR168 at levels higher than the Col wildtype control an equal and consistent production of both 21-nt and 22-nt miR168 species from the *MIR168b* stem-loop (Figure 3B).

**Figure 4:**
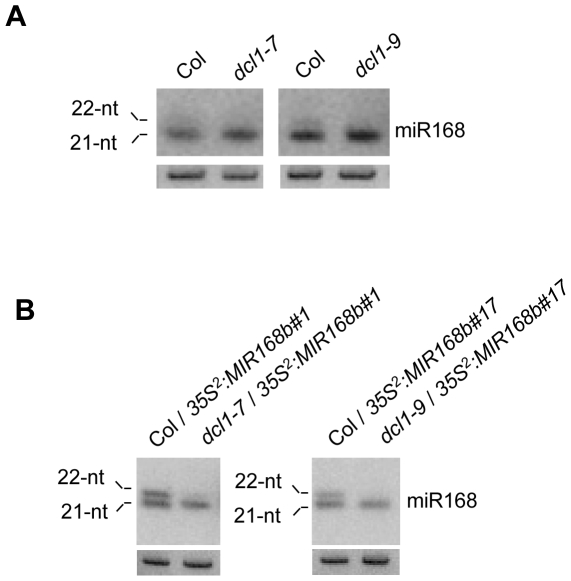
The 22-nt miR168 species is sensitive to *dcl1* mutations. (A) miR168 accumulation in Col and *dcl1-7* and *dcl1-9* mutants. RNA gel blot analysis of 10 µg of RNA with a probe complementary to miR168. U6 serves as a loading control. (B) miR168 accumulation in Col and *dcl1-7* or *dcl1-9* siblings deriving from the transformation of *dcl1-7/DCL1* or *dcl1-9/DCL1* heterozygotes by the *35S^2^∶MIR168b* construct in the binary vector pCambia1200. RNA gel blot analysis of 10 µg of RNA with a probe complementary to miR168. U6 serves as a loading control.

In wildtype plants, the 22-nt miR168 species often was masked by or merged with the signal of the abundant 21-nt species. However, high resolution northern blots allowed detection of the 22-nt miR168 species in wildtype plants (Figure 4A). Pyrosequencing revealed that the 22-nt TCGCTTGGTGCAGGTCGGGAAC miR168 species represents 5% of the overall cloned miR168 sequences from wildtype plants [20] and differs from the 21-nt species by the addition of a C at its 3′ end (Table 1). This 3′ C is present in both the *MIR168a* and *MIR168b* sequences, suggesting that the 22-nt species likely is a *bona fide* miRNA deriving from the two *MIR168* loci and does not result from the addition of a C after processing of the 21-nt species. Supporting this hypothesis, both 21- and 22-nt miR168a* and miR168b* sequences have been cloned [20]. The 21-nt miR168a* species was 40 times more abundant than the 22-nt miR168a* species whereas the 21-nt miR168b* species was only 4 times more abundant than the 22-nt miR168b* species (Table 1). Although different miRNA* molecules may have different stabilities, their cloning frequencies and the analysis of *35S^2^∶MIR168a* and *35S^2^∶MIR168b* transformants support the hypothesis that the *MIR168b* stem-loop is more prone to produce 22-nt miR168 species than the *MIR168a* stem-loop.

### 22-nt but not 21-nt miR168 is sensitive to *dcl1* mutations

Two features that distinguish 21-nt miR168 from other conserved miRNAs have been described previously: i) miR168 over-accumulates in *4m-AGO1* plants, and ii) miR168 appears insensitive to mutations in the miRNA-processing enzyme DCL1. Indeed, the level of miR168 is similar in wildtype Arabidopsis and *dcl1-7, dcl1-8* and *dcl1-9* hypomorphic mutants whereas these mutations negatively affect the accumulation of the other tested conserved miRNAs (Figure 3 and [28], [30]). The level of miR168 is unchanged in *dcl2, dcl3* and *dcl4* single mutants and in double, triple and quadruple *dcl* mutant combinations [30], indicating that miR168 is not processed by the other DCLs. Rather, these results suggest that AGO1 is limiting in wildtype plants and that miR168 competes with other miRNAs for incorporation into AGO1 [28]. In cases where AGO1 levels are not limiting, such as in a *dcl1* hypomorphic mutants in which the processing of many *MIRNA* precursors is impaired or in *4m-AGO1* plants where AGO1 levels are increased, more miR168 successfully associates with AGO1. As a result, the preferential stabilization of miR168 by the excess AGO1 in *dcl1* mutants likely masks the reduced processing of *MIR168* precursors.

Having revealed the production of a 22-nt miR168 species, the sensitivity of the 21-nt and 22-nt miR168 species to hypomorphic *dcl1* mutations was compared by RNA gel blot analysis. Whereas *dcl1* mutations do not impact the accumulation of 21-nt miR168, the 22-nt miR168 is below detectable levels in *dcl1-7* and *dcl1-9* mutants (Figure 4A). To confirm this distinct behavior of the two miR168 species, the *35S^2^∶MIR168b* construct was introduced into plants heterozygous for either the *dcl1-7* or *dcl1-9* mutation. In each mutant background, one *dcl1/DCL1* transformant showing detectable accumulation of the 22-nt miR168 species and insertion of the *35S^2^::MIR168b* construct at a single locus was analyzed further. After self-fertilization, isogenic *dcl1* and *DCL1* siblings were harvested by bulk, allowing a comparative analysis of miR168 accumulation in wildtype and mutant backgrounds. The accumulation of the 22-nt miR168 species was strongly reduced in *dcl1-7* and *dcl1-9* mutants, similar to the vast majority of conserved miRNAs, whereas the level of the 21-nt miR168 species remained unchanged (Figure 4B), suggesting that these two miR168 species are distinctly regulated.

### 21-nt but not 22-nt miR168 species are preferentially stabilized by AGO1

**Figure 5:**
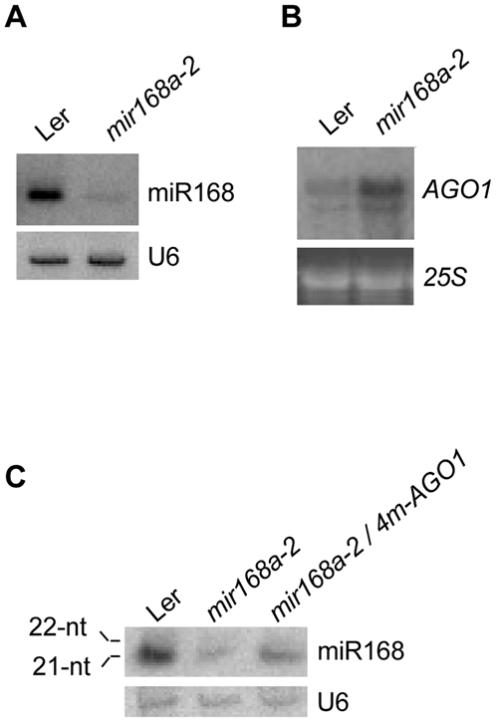
A *mir168a* loss-of-function mutant has reduced miR168 accumulation and increased *AGO1* levels. (A) miR168 accumulation in wildtype Ler and the *mir168a-2* mutant. RNA gel blot analysis of 10 µg of RNA with a probe complementary to miR168. U6 serves as a loading control. (B) *AGO1* mRNA accumulation in wildtype Ler and the *mir168a-2* mutant. RNA gel blot analysis of 10 µg of RNA with a T7-transcribed RNA probe complementary to *AGO1*. Ethidium bromide staining of 25S rRNA is shown as a loading control. (C) miR168 accumulation in wildtype Ler, the *mir168a-2* mutant and the *mir168a-2* transformed with the *4m-AGO1* construct. RNA gel blot analysis of 10 µg of RNA with a probe complementary to miR168. U6 serves as a loading control.

To further characterize the behavior of the 21- and 22-nt miR168 species, *mir168a* and *mir168b* mutants were analyzed. Likely due to the presence of a 35S promoter within the T-DNA inserted in the *MIR168a* and *MIR168b* promoters, *mir168a-1d* (SALK_113514) and *mir168b-1d* (SALK_066855) mutants accumulated miR168 to levels slightly higher than wildtype plants and did not exhibit obvious developmental defects ([28]; data not shown). Loss-of-function *mir168b* mutants were not available in the public databases. In contrast, the *mir168a-2* mutant (CSHL_GT305) carries a Ds element inserted between the transcription start and the miRNA stem-loop and miR168 accumulation was reduced to 15% of the wildtype level (Figure 5A). This result is consistent with the *pMIR168a∶GUS* fusion being more strongly expressed than the *pMIR168b∶GUS* fusion (Figure 2) and with miR168a* being more frequently cloned than miR168b* (Table 1) and indicates that the majority of miR168 derives from the *MIR168a* locus. As a consequence of the decrease in miR168 production in the *mir168a-2* mutant, *AGO1* mRNA levels were increased by three-fold, revealing that the *MIR168b* locus *per se* is insufficient to maintain AGO1 homeostasis, at least at the mRNA level (Figure 5B).

Because an equal amount of 21-nt and 22-nt miR168 species is observed in *35S^2^∶MIR168b* transformants in which miR168 is predominantly produced by the *35S^2^∶MIR168b* transgene (Figure 3A), the *mir168a-2* mutant, which produces miR168 only from the *MIR168b* locus, was expected to produce an equal amount of 21-nt and 22-nt miR168 species. However, only the 21-nt miR168 was detected in the *mir168a-2* mutant (Figure 5A). The fact that *35S^2^∶MIR168b* transformants with lower than wildtype AGO1 levels exhibit a lower 21/22 ratio than wildtype plants whereas the *mir168a-2* mutant with higher than wildtype AGO1 levels exhibits a higher 21/22 ratio than wildtype plants suggests that preferential stabilization of miR168 by AGO1 only affects the 21-nt miR168 species but not the 22-nt species. Supporting this hypothesis, expression of the *4mAGO1* transgene in the *mir168a-2* mutant led to increased levels of the 21-nt miR168 species compared to the *mir168a-2* mutant but did not affect 22-nt miR168 accumulation (Figure 5C).

### *mir168a-2* mutants exhibit developmental defects

**Figure 6:**
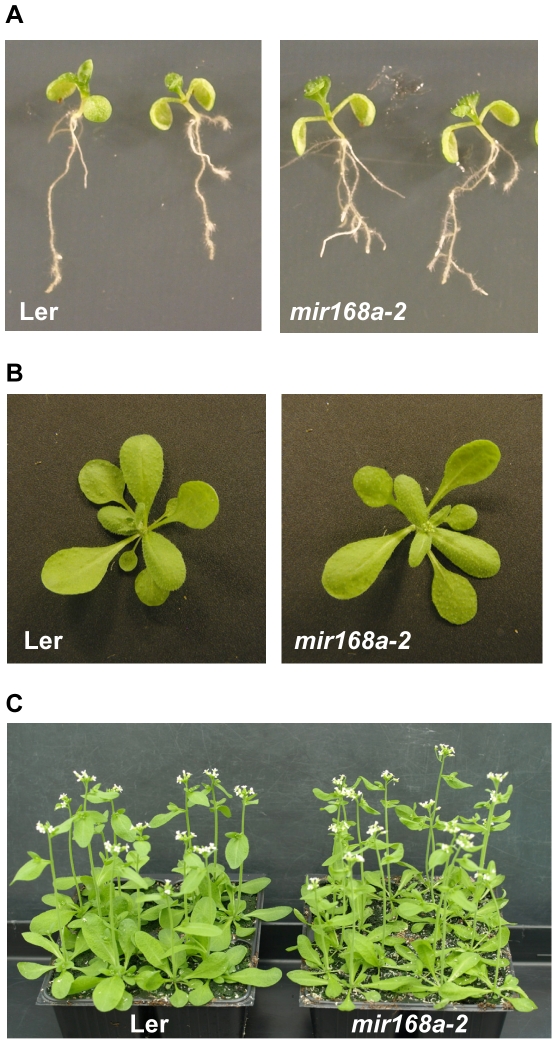
A *mir168a* loss-of-function mutant exhibits developmental defects. (A) Ten-day-old wildtype Ler and the *mir168a-2* mutant sown in vitro on vertical plates and grown in a controlled growth chamber at 18°C. (B) Twenty-two-day-old wildtype Ler and the *mir168a-2* mutant sown directly on soil and grown in a controlled growth chamber at 22°C. (C) Twenty-five-day-old wildtype Ler and the *mir168a-2* mutant sown directly on soil and grown in a glasshouse where temperature varies between a minimum of 13°C at night and a maximum of 30°C during the day.

When grown *in vitro* on vertically-oriented plates, the *mir168a-2* mutant consistently exhibited an increased number of lateral roots compared with wildtype plants grown on the same plates (Figure 6A), consistent with a previous report showing that *ago1* null alleles have a decreased number of lateral roots [31]. Aerial parts of the *mir168a-2* mutant did not exhibit obvious developmental defects when grown *in vitro* or when sown directly on soil and grown in a growth chamber under standard controlled conditions (Figure 6B). However, high temperature promoted early flowering of *mir168a-2* compared with wildtype plants (Table 2). Moreover, when grown in a glasshouse where plants are exposed to abiotic stresses such as temperature, light and water fluctuations, *mir168a-2* mutants were less vigorous and consistently exhibited narrow, twisted leaves and flowered earlier than wildtype plants (Figure 6C). Altogether, these results indicate that the *MIR168b* locus is not sufficient for proper development, particularly during environmentally challenging conditions.

**Table 2:** Flowering characteristics of the *mir168a-2* mutant.

Growth conditions	Ler (DAG)	*mir168a-2* (DAG)
In vitro 18°C	28	29
In vitro 24°C	23	19
Soil growth chamber 22°C	27	24
Soil glasshouse 13–30°C	22	19
15 days in vitro 18°C ->soil growth chamber	28	27
15 days in vitro 18°C ->soil glasshouse	26	25

DAG indicates the number of days after germination at which 50% of the plants had open flowers.

## Discussion

It was recently shown that the association of small RNAs with different AGO proteins partly depends on the nature of their 5′ nucleotide. Indeed, miRNAs that have a 5′U, 5′A or 5′C associate with AGO1, AGO2 and AGO5, respectively, whereas 24-nt siRNAs, which predominantly have a 5′A, associate with AGO4 [12], [13], [32]. However, there are other associations that remain unexplained. For example, AGO7 exclusively associates with miR390, which has a 5′A [12]. In addition, some miRNAs likely are able to associate with other AGO in the absence of AGO1. Indeed, while the accumulation of most miRNAs is strongly reduced in a null *ago1* mutant, the levels of miR156/157 and miR167 are unchanged [11]. It is likely that additional proteins influence the incorporation and/or stabilization of miRNA into AGO proteins. For example, *4m-AGO1* plants that have increased *AGO1* levels exhibit unchanged accumulation of most miRNAs but strongly increased accumulation of miR168 and to some extent miR159 and miR165/166 [28]. Such a preferential stabilization of miR168 by AGO1, in conjunction with miR168-guided *AGO1* mRNA cleavage and transcriptional co-regulation of *AGO1* and *MIR168* genes, maintains AGO1 homeostasis [28].

Pyrosequencing-based counting of miR168, miR168a* and miR168b* molecules in wildtype plants [20] and northern analysis of the null *mir168a-2* mutant revealed that *MIR168a* contributes the majority of miR168 molecules and likely is sufficient for plants to develop normally. Supporting this hypothesis, a mutagenized *4m-MIR168a* gene expressing a compensatory miRNA rescues developmental defects triggered by a *4m-AGO1* gene. By contrast, the *MIR168b* locus appears to contribute much less miR168 molecules than *MIR168a*. Nevertheless, a mutagenized *4m-MIR168b* gene expressing the same compensatory miRNA also rescues developmental defects triggered by *4m-AGO1* expression, and the *mir168a-2* mutant develops normally under non-stressful conditions. However, the *mir168a-2* mutant, which carries only a *MIR168b* locus, does not develop normally when stressed in a glasshouse, suggesting a role for *MIR168a* during stress adaptation. Whether the *MIR168b* locus is dispensable for proper development awaits the identification of a null *mir168b* mutant.

Several characteristics of the *MIR168b* locus distinguish it from the *MIR168a* locus, suggesting that *MIR168a* and *MIR168b* have specialized functions. *MIR168a* predominantly produces a 21-nt miR168 species whereas *MIR168b* produces an equal amount of 21- and 22-nt miR168 species, which differ by one nucleotide at their 3′ end [20]. The alternative processing at the 3′ end of the *MIR168b* stem-loop RNA precursor could occur due to the different structural features of the *MIR168a* and *MIR168b* stem-loops. Whereas pairing within the *MIR168a* RNA precursor stem extends beyond the 3′ end of miR168 sequence, the *MIR168b* precursor has a large bulge at the 3′ end of the miR168 sequence [19]. The 22-nt miR168 species differs from the 21-nt miR168 species in that it is not preferentially stabilized by AGO1 and consequently is more sensitive to *dcl1* mutations than the 21-nt species. This result suggests that the length or the 3′ nucleotide of miR168 influences its incorporation or stabilization by AGO1, raising the possibility that the length or 3′ nucleotide identity of other miRNAs could similarly affect their stability.

The existence of two *MIR168* loci that produces different levels of 21- and 22-nt miR168 species suggests that the fine tuned regulation of AGO1 homeostasis requires the combinatory action of the *dcl1*-sensitive AGO1-insensitive 22-nt miR168, which is produced at low levels, primarily by *MIR168b*, and the *dcl1*-insensitive AGO1-sensitive 21-nt miR168, which is produced at high levels, primarily by *MIR168a*. Indeed, *mir168a* mutants that have a drastically reduced level of 21-nt miR168 exhibit developmental defects, particularly during environmentally challenging conditions, suggesting that the high level of 21-nt miR168 provided by the *MIR168a* locus is essential for proper development. In conclusion AGO1 homeostasis likely requires the production of a low level of 22-nt miR168 to ensure basal cleavage of *AGO1* mRNA and a high level of 21-nt miR168 to allow an effective response to endogenous or environmental fluctuations owing to its post-transcriptional stabilization by AGO1.

## Materials and Methods

### Plant material

The *dcl1-7* and *dcl1-9* mutants [33] have been back-crossed four times to Col. The Salk Institute Genomic Analysis Laboratory [34] generated the sequence-indexed T-DNA insertion line SALK_066855 and the Cold Spring Harbor Laboratory [35] generated the sequence-indexed T-DNA insertion line CSHL_GT305.

### Molecular cloning and plant transformation

The *4m-AGO1*, *4m-MIR168a*, *35S^2^∶MIR168a* and *pMIR168a∶GUS* constructs have been described before [11], [28].

The *4m-MIR168b* construct was made as follows: The *MIR168b* gene was sub-cloned from BAC K9E15 into the Bluescript vector pKS+ as a 930 bp fragment (position 39950–40880 on K9E15). Compensatory mutations that restore complementarity to the *4m-AGO1* mRNA were introduced into the *MIR168b* gene using the Quick Change Site-Directed Mutagenesis Kit (Stratagene) . The *miR168* sequence was first mutageneized using the following pair of primers: 5′-cgtgtcggtgtcagccaatcagttcccgacctgcaccaagcgaatccgagaccgccggtaac-3′ and 5′-cgtgtcggtgtcagccaatcagtgccggacctccaccaggcgaatccgagaccgccggtaac-3′. The miR168* sequence was subsequently mutageneized using the following pair of primers : 5′-cacctcggactccgattcagctgatagaagaccggcgctcacaaaccaaccatgacaagacacg-3′ and 5′-cgtgtcttgtcatggttggtttgtgagcgccggtcttctatcagctgaatcggagtccgaggtg-3′. The mutagenized fragment was sequenced to ensure that no other mutations have been introduced and transferred from pKS+ into the pCambia1200 binary vector.

The *35S^2^∶MIR168b* construct was made as follows: The *MIR168b* region homologous to the *MIR168a* EST H77185 was amplified as a 435-nt fragment (position 40001–40435 on BAC K9E15) and cloned between the SalI and EcoRI sites in the pLBR19 vector. The *p35S^2^∶MIR168b:t35S* fragment was excised as a KpnI-XbaI and cloned between the KpnI and XbaI sites in the pBin+ or pCambia1200 binary vectors.

The construct *pMIR168b∶GUS* is a transcriptional fusion with a 479 bp fragment of the *MIR168b* gene (position −516–37 relative to the beginning of the hairpin). These nucleotide positions were chosen so as to exclude an ATG located at position −29 that could compromise the use of the GUS reporter initiation codon and to keep the entire promoter region previously used to express the compensatory miRNA 4m-miR168 from a *4m-MIR168b* transgene, which rescued the 4m-AGO1 phenotype. To built *pMIR168b∶GUS*, the *MIR168b* promoter was amplified from the *4m-MIR168b* construct in pKS+ as a 474-nt fragment (position 40407–40880 on BAC K9E15) and cloned in the pBI101.2 binary vector.

All constructs were transferred to Agrobacterium tumefaciens by triparental mating and Arabidopsis plants were transformed using the floral dip method [36]. Collected seeds were surface sterilized and transformants were selected by plating seeds on a medium supplemented with either 50 µg/ml kanamycin (pBin+) or 30 µg/ml hygromycin (pCambia1200). Plants were grown under cool-white light in long days (16 h of light, 8 h of dark) at 23°C.

### RNA analysis

Total RNA was isolated from rosette leaves as described [11].

RNA gel blot analysis of low molecular weight RNAs was carried out on denaturing 15% polyacrylamide gels, followed by blotting to a nylon membrane (Genescreen Plus, PerkinElmer Inc) as described [11]. Blots were hybridized with gamma-ATP ^32^P end-labeled oligonucleotides. Blots were re-hybridized with an end-labeled oligonucleotide probe complementary to U6.

For mRNA gel blot analysis, RNA was separated on denaturing 1% agarose gels and trasferred to nylon membrane (Genescreen Plus, PerkinElmer Inc). Blots were hybridized with an alpha-UTP ^32^P labeled RNA probe complementary to *AGO1* mRNA in ULTRAhyb buffer at 68°C (Ambion). AGO1 RNA probes were generated by PCR using primers 5′-CTACAGGGATGGAGTCAGTGAGGGAC-3′, 5′-GGCCGTAATACGACTCACTATAGGCTCCCACTAGCCATTGAGCCACTG-3′ followed by T7-mediated in vitro transcription.
